# Reliability of corticospinal excitability and intracortical inhibition in biceps femoris during different contraction modes

**DOI:** 10.1111/ejn.15868

**Published:** 2022-12-07

**Authors:** Joel D. Presland, Paul J. Tofari, Ryan G. Timmins, Dawson J. Kidgell, David A. Opar

**Affiliations:** ^1^ School of Behavioural and Health Sciences Australian Catholic University Melbourne Victoria Australia; ^2^ Sports Performance, Recovery, Injury & New Technologies (SPRINT) Research Centre Australian Catholic University Melbourne Victoria Australia; ^3^ School of Primary and Allied Health Care Monash University Melbourne Victoria Australia

**Keywords:** hamstring, intracortical facilitation, intracortical inhibition, isokinetic dynamometry, transcranial magnetic stimulation

## Abstract

This study aimed to determine the test–retest reliability of a range of transcranial magnetic stimulation (TMS) outcomes in the biceps femoris during isometric, eccentric and concentric contractions. Corticospinal excitability (active motor threshold 120% [AMT120%] and area under recruitment curve [AURC]), short‐ and long‐interval intracortical inhibition (SICI and LICI) and intracortical facilitation (ICF) were assessed from the biceps femoris in 10 participants (age 26.3 ± 6.0 years; height 180.2 ± 6.6 cm, body mass 77.2 ± 8.0 kg) in three sessions. Single‐ and paired‐pulse stimuli were delivered under low‐level muscle activity (5% ± 2% of maximal isometric root mean squared surface electromyography [rmsEMG]) during isometric, concentric and eccentric contractions. Participants were provided visual feedback on their levels of rmsEMG during all contractions. Single‐pulse outcomes measured during isometric contractions (AURC, AMT110%, AMT120%, AMT130%, AMT150%, AMT170%) demonstrated fair to excellent reliability (ICC range, .51 to .92; CV%, 21% to 37%), whereas SICI, LICI and ICF demonstrated good to excellent reliability (ICC range, .62 to .80; CV%, 19 to 42%). Single‐pulse outcomes measured during concentric contractions demonstrated excellent reliability (ICC range, .75 to .96; CV%, 15% to 34%), whereas SICI, LICI and ICF demonstrated good to excellent reliability (ICC range, .65 to .76; CV%, 16% to 71%). Single‐pulse outcomes during eccentric contractions demonstrated fair to excellent reliability (ICC range, .56 to .96; CV%, 16% to 41%), whereas SICI, LICI and ICF demonstrated good to excellent (ICC range, .67 to .86; CV%, 20% to 42%). This study found that both single‐ and paired‐pulse TMS outcomes can be measured from the biceps femoris muscle across all contraction modes with fair to excellent reliability. However, coefficient of variation values were typically greater than the smallest worthwhile change which may make tracking physiological changes in these variables difficult without moderate to large effect sizes.

**Abbreviations**
AMT: active motor threshold
AURC: area under recruitment curve
ICC: intraclass correlation coefficient
ICF: intracortical facilitation
ISI: interstimulus interval
LICI: long‐interval intracortical inhibition
MEP: motor evoked potential
MVC: maximum voluntary contraction
PPA: peak‐to‐peak amplitude
rmsEMG: root mean square of surface electromyography
sEMG: surface electromyography
SICI: short‐interval intracortical inhibition
TMS: transcranial magnetic stimulation

## 1 INTRODUCTION

Transcranial magnetic stimulation (TMS) is a non‐invasive method of brain stimulation used to investigate the excitatory and inhibitory circuits within the primary motor cortex (M1) which project onto the corticospinal tract (Chen, 2000). This is conducted by passing a current through an electromagnetic coil placed over the cortical representation of the muscle of interest to produce a varying magnetic field (Chen, 2000). During M1 stimulation, the changing magnetic field induces electrical currents via axonal depolarisation of neurons which synapse onto corticospinal neurons that innervate skeletal muscle (Carroll et al., 2001). Induced action potentials in cortical axons spread trans‐synaptically to connected cortical and subcortical regions, creating a volley of excitation along the corticospinal tract and peripheral motor nerve, resulting in a response at the muscle (Groppa et al., 2012). The electrical response (motor evoked potential [MEP]), measured at the target muscle using surface electromyography (sEMG), forms the basis of TMS outcomes (Hallett, 2000).

Using single‐pulse stimulation, the peak‐to‐peak amplitude (PPA) of elicited MEPs can be used to measure corticospinal excitability (Chipchase et al., 2012). Stimulus–response curves can be constructed using a range of single‐pulse stimulation intensities to measure the excitability of the target muscle's motor representation (Iyer & Madhavan, 2019). Paired‐pulse TMS provides insight into the influence of inhibitory and facilitatory inputs of the M1 on the corticospinal tract (Chen, 2000). The paired‐pulse outcomes, short‐interval intracortical inhibition (SICI) and long‐interval intracortical inhibition (LICI), allow for the quantification of intracortical inhibitory input mediated by gamma aminobutyric acid‐a (GABA‐a) (McDonnell et al., 2006) and Gamma aminobutyric acid‐b (GABA‐b) receptors (Rogasch et al., 2009), respectively. Conversely, this method can also be used to conduct measurements of intracortical facilitation (ICF) which may assess glutamate mediated excitation within circuits of the M1 (Chen, 2004). However, subcortical mechanisms have also been shown to contribute to ICF in the upper limb (Wiegel et al., 2018).

The TMS outcomes measured at the target muscle exhibit within‐subject variability. A range of factors have been shown to influence the variance of TMS outcomes including variability in the location and the orientation of the TMS coil (Carroll et al., 2001), poor replication of sEMG electrode positioning (Carroll et al., 2001), changes in skin impedance (Hermens et al., 2000) or the skin–electrode interface (Hermens et al., 2000) as well as the constant oscillation in corticospinal neuron excitability (Kiers et al., 1993). Despite such sources of variation, satisfactory test–retest reliability has been found in lower limb muscles including the quadriceps (Leung et al., 2018; O'Leary et al., 2015; Sidhu et al., 2009; Temesi et al., 2017), gastrocnemius (Fisher et al., 2014) and tibialis anterior (Fisher et al., 2014; Tallent et al., 2012; van Hedel et al., 2007). However, the majority of literature examining lower limb corticospinal excitatory and inhibitory properties using TMS have been conducted under resting conditions or during low‐intensity isometric muscle contractions (O'Leary et al., 2015; Sidhu et al., 2009; Temesi et al., 2017; van Hedel et al., 2007) with only one study investigating these measures during concentric and eccentric contractions in the lower limb (tibialis anterior) (Tallent et al., 2012). Different contraction modes (i.e., concentric, eccentric and isometric) are shown to exhibit distinct corticospinal control patterns in the lower limb (Duclay et al., 2011), and a greater understanding of the reliability of assessing excitatory and inhibitory properties under different contraction modes in lower limb muscles is required.

A greater focus on concentric and eccentric contractions may be particularly relevant for the application of TMS to specific muscle groups or populations. For example, those with a history of hamstring strain injury display deficits in hamstring strength and biceps femoris (the most commonly injured of all the hamstring muscles) sEMG activity during maximal eccentric contractions (Opar et al., 2013). However, these deficits are absent during maximal concentric contractions (Opar et al., 2013). Such deficits are noteworthy as a submaximally stimulated lengthening muscle can absorb less energy prior to stretch induced failure *(in‐situ)* (Mair et al., 1996), and *in‐vivo* strain injuries are associated with high‐force eccentric contractions (Chumanov et al., 2007). Therefore, determining the reliability of corticospinal responses in the hamstrings within each contraction mode, rather than just isometric, is of importance. Comparing TMS elicited MEPs in the hamstrings across each mode of contraction is of interest given prior investigations of altered spinal and supraspinal control of neuromuscular activity during eccentric contractions in other lower limb muscles such as the gastrocnemius and soleus (Aagaard, 2018). However, there is no literature investigating the reliability of single‐ or paired‐pulse TMS outcomes within any contraction mode from the hamstring muscle group.

While the reliability of TMS outcomes has been established in other lower limb muscle groups (Fisher et al., 2014; Leung et al., 2018; O'Leary et al., 2015; Sidhu et al., 2009; Tallent et al., 2012; Temesi et al., 2017; van Hedel et al., 2007), the test–retest reliability of these outcomes vary, even across synergistic muscle groups (Malcolm et al., 2006). It is important to establish the reliability of TMS outcomes to ensure measurement of physiological parameters are not adversely affected by measurement error or variation in factors such as sEMG or coil placement. Therefore, the aim of this study was to assess the test–retest reliability of a range of TMS outcomes from the biceps femoris during isometric, eccentric and concentric contractions.

## 2 METHODS

### 2.1 Participants

Fifteen recreationally active male participants were recruited for this study. In five of these participants, an MEP was not able to be elicited despite extensive delivery of TMS across the cortex at various locations and stimulus intensities, resulting in a final sample of 10 participants (age 26.3 ± 6.0 years; height 180.2 ± 6.6 cm, body mass 77.2 ± 8.0 kg). All participants were free of any major lower limb injury in the previous 36 months as well as having no history of neurological diseases (e.g., epilepsy and Parkinson's). All participants provided informed written consent prior to participating in the study, which was undertaken at Australian Catholic University, Fitzroy, Victoria, Australia. Ethical approval for this study was granted by the Australian Catholic University Human Research Ethics Committee (approval number 2018‐281H).

### 2.2 Study design

**FIGURE 1:**
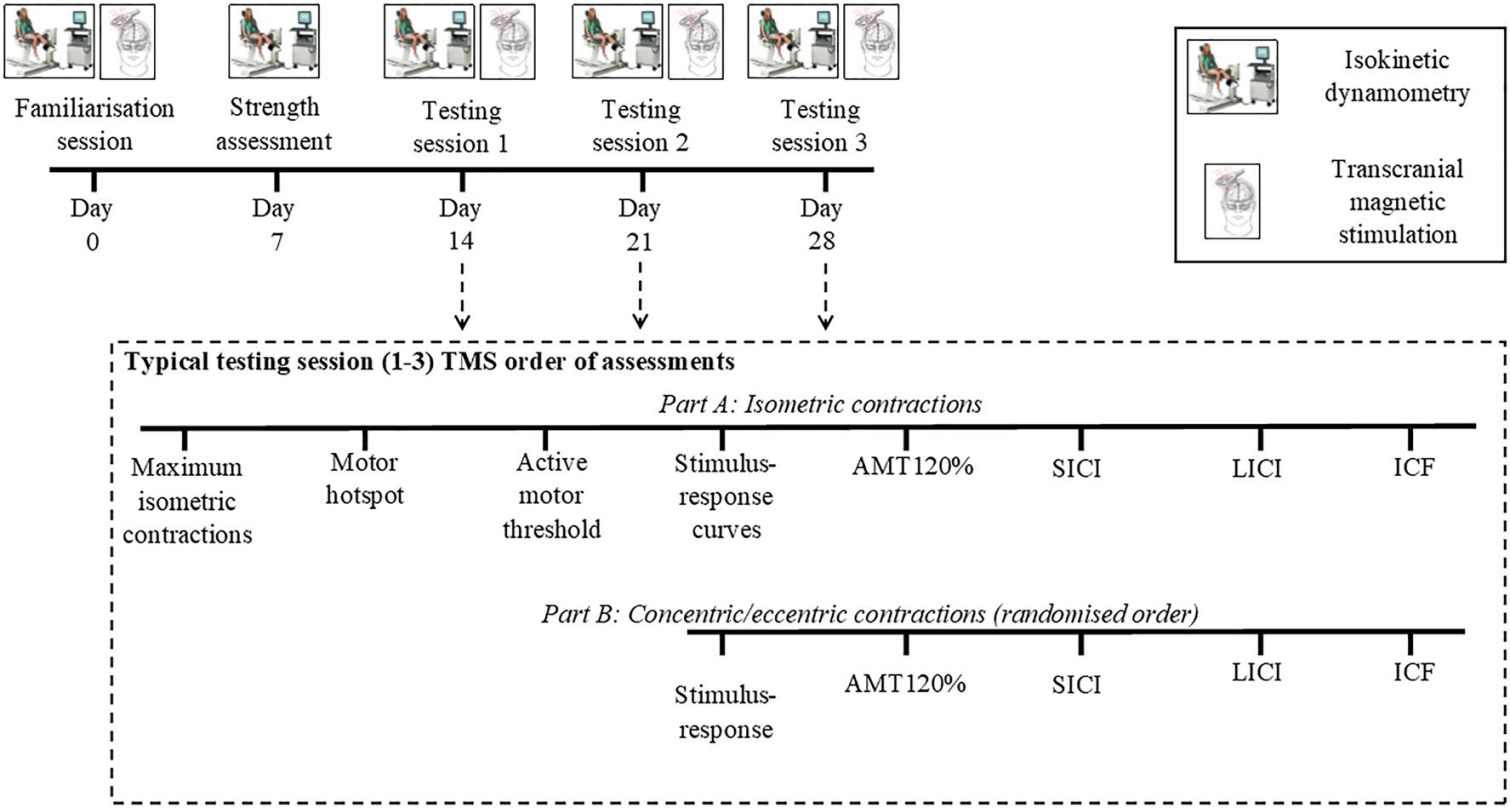
Schematic representation of the experimental design and completion order of transcranial magnetic stimulation outcomes during testing sessions 1 to 3. Note that the days between sessions is representative only and does vary slightly between participants. Part A refers to stimuli delivered during isometric contractions, whereas part B refers to stimuli delivered during concentric and eccentric contractions. TMS, transcranial magnetic stimulation; AMT120%, single‐pulse TMS at 120% of active motor threshold stimulator output; SICI, short‐interval intracortical inhibition; LICI, long‐interval intracortical inhibition; ICF, intracortical facilitation. Stimulus response curves = sEMG responses collected across all contraction modes by delivering stimuli at each of 110%, 130%, 150% and 170% of AMT. SICI was determined using a 3 ms interstimulus interval (ISI) and conditioning and test stimuli of 80% and 120% of AMT. LICI was determined using an ISI of 100 ms and conditioning and test stimuli intensities of AMT120%. ICF was quantified using the same conditioning and test stimuli as used during SICI with an ISI of 12 ms.

A schematic representation of the testing sessions completed and the order of outcome testing within them can be found in Figure 1. Participants undertook a familiarisation session prior to their first testing session. Participants were familiarised to single‐ and paired‐pulse TMS, isometric, concentric and eccentric maximum voluntary contractions (MVCs) of the knee flexors. At least 3 days after the familiarisation session (mean 7.3 ± 4.8 days), participants had their MVC strength assessed during all contraction modes using an isokinetic dynamometer. All assessments were conducted unilaterally on the participant's dominant limb (defined as the preferred leg used for kicking). At least seven days (mean 9.8 ± 3.4 days) following strength testing, all participants completed their first TMS testing session (Session 1) where they received a series of single‐ and paired‐pulse stimulations while maintaining low‐intensity isometric, concentric and eccentric knee flexor contractions (5% ± 2% of root mean squared sEMG [rmsEMG] during maximal isometric contraction) to assess corticospinal function. This session was repeated in an identical manner on two subsequent occasions (Sessions 2 and 3, respectively), with at least 3 days between sessions (mean 8.0 ± 3.3 days). All familiarisation and testing sessions were conducted at the same time of day for each participant.

### 2.3 Outcome measures

#### 2.3.1 Isokinetic dynamometry

All contractions were completed on an isokinetic dynamometer (Biodex System 4, Medical Systems Inc, New York, USA), and torque data were collected at a frequency of 1 kHz using custom software (LabVIEW 2017 National Instruments, Austin, Texas). Participants were seated on a custom‐made pad placed on top of the dynamometer which had cut out sections at the approximate location of the electrodes to minimise sEMG noise due to contact with the seat. The hip was flexed at approximately 85° from full extension (0° = neutral hip position). Dynamometer position was set up and recorded during the first session and replicated for all subsequent sessions. Straps across the chest, waist, distal thigh and shank (superior to lateral malleolus) were used to minimise extraneous movement and to maintain alignment of the knee joint axis of rotation with the fulcrum of the dynamometer lever arm. Limb weight gravity correction was subsequently conducted with the limb fixed at 30° knee flexion (full extension = 0°).

Isometric muscle contractions of the knee flexors were performed at 30° knee flexion (full extension = 0°). Participants were instructed to ‘pull down’ against the lever as hard and fast as possible and to maintain this contraction until a verbal cue was given to cease the attempt (approximately 5 s). For concentric and eccentric knee flexor muscle contractions, the range of motion was set between 10° and 50° of knee flexion. Each eccentric contraction began at 50° of knee flexion whilst concentric contractions started at 10° of knee flexion. Isokinetic concentric and eccentric contractions were performed at 10°·s^−1^ during strength testing and corticospinal function assessment sessions.

#### 2.3.2 Strength testing sessions

Maximum voluntary strength testing of the knee flexors was conducted following three isometric warm up contractions performed at 50%, 75% and 95% of a perceived maximum effort. Knee flexor isometric strength testing was performed first, followed by either a concentric or eccentric knee flexor contraction chosen at random to reduce order effects between contractions. Participants were given visual feedback of their efforts and encouraged verbally by instructors to ensure a maximal effort. Between each maximal effort, participants had a 60 s rest period. The greatest torque output detected from the three efforts was used to determine the participant's peak torque for each contraction mode. Additional efforts were performed if torque in the final repetition was 5% greater than the highest value recorded from the preceding trials.

#### 2.3.3 Maximal voluntary contractions for normalisation of sEMG data

Three isometric contractions were performed at 50%, 75% and 95% of the participant's perceived maximal intensity at the beginning of each corticospinal function assessment sessions (sessions 1 to 3). After this, three isometric MVCs of the knee flexors were completed to obtain the maximal rmsEMG value of the biceps femoris sEMG. The rmsEMG was used to determine the intensity of effort required during all TMS contractions for each individual session.

#### 2.3.4 sEMG

Bicep femoris sEMG data was obtained via circular bipolar pre‐gelled DUO‐TRODE Ag/AgCl electrodes (NAOL Australia, NSW, Australia; interelectrode distance = 21 mm) and was collected using a wireless EMG system (Myon m320RX/TX, Schwarzenberg, Switzerland). Electrode placement was performed according to the SENIAM guidelines (Hermens et al., 2000), where the area of electrode placement was shaved, abraded and cleaned with a 70% isopropyl alcohol wipe. Biceps femoris electrodes were placed at 50% of the line between the ischial tuberosity and the lateral and medial epicondyles of the tibia, respectively.

#### 2.3.5 TMS

Stimulations were manually delivered via TMS using two Magstim 200^2^ (Carroll et al., 2001) stimulators connected via a Bi‐stim unit while using a double‐cone coil (110 mm external loop diameter; Magstim Co, The Magstim Company, Whitland, UK). The coil was placed on the contralateral hemisphere (Buhmann et al., 2022) to induce MEPs in the biceps femoris on the dominant leg (i.e., the left hemisphere was stimulated to elicit MEPs in the right biceps femoris). All stimuli were applied during low‐level muscle activity which equated to 5% ± 2% of maximal isometric rmsEMG recorded during the same session. This level of pre‐stimulus background electromyography has been utilised previously in upper limb TMS literature (Sale & Semmler, 2005). Visual feedback of rmsEMG was provided to participants as a percentage of their maximal rmsEMG. Participants were familiarised to and allowed practice in reaching the 5% rmsEMG target in each contraction mode during each session. Any repetition outside of the 5% target by ±2% rmsEMG was discarded and repeated.

Participants wore a fitted cap (Textile caps, Sonoray, Adelaide, Australia) which was marked with a latitude–longitude matrix (1 cm intervals) positioned in reference to interaural and nasion–inion lines (Kidgell et al., 2015; Pearce et al., 2013). Grid placement was guided by the estimated position of M1 cortical representation of the hamstrings, which is located at the midline of the cerebral cortex close to the interhemispheric fissure (Groppa et al., 2012). The optimal site of stimulation (a.k.a. motor hotspot) was determined as the site of the largest mean PPA MEP (of three stimulations at each site) (Pearce et al., 2013) detected from the biceps femoris muscle following a systematic mapping and stimulation of various cortical coordinates. A stimulus intensity of 60% of maximal stimulator output was typically used for determining the motor hotspot, however, participants that responded with PPA <500μV received larger stimulation intensities during hotspot determination. All subsequent stimulations were delivered over the hotspot site. Active motor threshold (AMT) was established as the stimulator intensity where at least five of 10 stimuli resulted in MEPs with a PPA of ≥500μV (Adank et al., 2018). Determination of AMT was conducted in a stepwise manner of 2% to 5% decrements of stimulator intensity (Pearce et al., 2013), starting at 60% of maximum stimulator output. Both motor hotspot and AMT determination were conducted during isometric contractions only and the resulting stimulation intensity and location were utilised during corticospinal function assessment for all contraction modes, as per previous work (Kidgell et al., 2015). During concentric and eccentric contractions, stimulations were delivered while the limb was contracting and at 30° of knee flexion. For all stimulations delivered during isometric contractions (including during AMT and motor hotspot assessments), the limb was fixed at 30° knee flexion.

Single‐pulse stimulus–response curves were collected across all contraction modes by delivering stimuli at each of 110%, 130%, 150% and 170% of AMT (Frazer et al., 2019). Stimuli delivered at AMT120% were used to measure corticospinal excitability and were undertaken after the stimulus–response curve assessments were completed.

Quantification of SICI was conducted utilising a 3 ms interstimulus interval (ISI) and conditioning and test stimuli at 80% and 120% of AMT, respectively (Latella et al., 2016). LICI was determined using an ISI of 100 ms and conditioning and test stimuli intensities of AMT120% (Kidgell et al., 2015; Latella et al., 2016). ICF was quantified using the same conditioning and test stimuli as used during SICI with an ISI of 12 ms (Latella et al., 2016).

For concentric or eccentric contractions, each reported test type (i.e., AMT110%) received 10 to 20 stimulations, with the first 10 trials which adhered to the following criteria being included in analysis: (a) The stimulation was delivered in the acceptable range of motion, and (b) the stimulation was delivered while pre‐stimulus rmsEMG was 5% ± 2% of maximal isometric rmsEMG. Trials that did not adhere to these criteria were discarded and repeated until 10 acceptable trials were collected.

### 2.4 Data analysis

#### 2.4.1 sEMG data

All sEMG data were recorded in millivolts (mV) and collected using custom software (LabVIEW 2017 National Instruments, Austin, Texas) at a frequency of 1 kHz. Raw sEMG data were filtered using a zero‐lag fourth‐order Butterworth filter with a high‐pass frequency of 13 Hz and a low‐pass frequency at 500 Hz. All EMG data were normalised to the largest rmsEMG value obtained during a maximal isometric knee flexion contraction during the same session (MEP PPA/maximal isometric rmsEMG). Pre‐stimulus rmsEMG was defined as the 100 ms epoch prior to stimulation. For each testing variable within a session, the EMG‐time data for all 10 repetitions were averaged and then used for further analysis.

#### 2.4.2 Corticospinal excitability, intracortical inhibition and facilitation data

The PPA of MEPs was automatically detected using custom software (LabVIEW 2017; National Instruments, Austin, Texas) as the difference between the maximum and minimum value in the 100 ms epoch following stimulation, which was subsequently confirmed via visual inspection. Single‐pulse stimulus–response curves were derived by plotting stimulus intensity (110%, 130%, 150% and 170% of AMT) against normalised MEP amplitude, and the total area under the recruitment curve (AURC) was calculated using the trapezoidal integration method (Carson et al., 2013).

With respect to paired‐pulse measures, LICI was calculated as the ratio between the PPAs of the test stimuli response and the conditioning stimuli response (test stimuli MEP PPA/conditioning stimuli MEP PPA) (McNeil et al., 2011). A ratio of the AMT120% MEP and test stimuli MEP (test stimuli MEP PPA/AMT120% MEP PPA) was used to calculate SICI. ICF was calculated as the ratio between the test stimuli MEP and the AMT120% MEP obtained during single‐pulse TMS (test stimuli MEP PPA/AMT120% MEP PPA).

#### 2.4.3 Statistical analysis

All statistical analysis was performed using a custom spreadsheet which determined the test–retest reliability of all TMS outcomes (Hopkins, 2017). The extent of variation between each test pulse intensity in the AURC (110%, 130%, 150% and 170% of AMT) and corticospinal function assessment (AMT120%, SICI, LICI and ICF) was determined from Sessions 1 to 2 and Sessions 2 to 3, respectively. This variation was quantified by calculating typical error (TE), intraclass correlation coefficients (ICC) (3, 1) (Hopkins, 2000) and TE as a coefficient of variation (CV%). Guided by prior TMS literature (Temesi et al., 2017), an ICC value of <.40 was subjectively interpreted as poor, between .40 and .59 was fair, .60 to .74 was good and ≥.75 was excellent. Minimal detectable change at a 95% confidence interval (MDC_95_) was calculated as TE × 1.96 × √2. The smallest worthwhile change (SWC) of each outcome was calculated as .2 × between‐subject standard deviation. Sample size calculations (Arifin, 2022) were guided by earlier TMS literature investigating between‐session reliability. This was largely guided by the ICCs reported in other TMS literature summarised in a systematic review (Cavaleri et al., 2017). Given studies included in this review utilising a similar amount of stimulations (5–15) reported ICCs of .81 to .98, a ICC value of .80 was used in these calculations. Other input variables include a precision of .20, a confidence level of 95%, the number of raters/repetitions per subject was 3 and the expected dropout rate was 10%. This calculation anticipated 10 participants would be required to have sufficient power given these inputs.

## 3 RESULTS

The mean duration of time between Sessions 1 to 2 and 2 to 3 was 8.6 ± 4.0 and 7.4 ± 2.4 days. In one participant, who had successfully completed Sessions 1 and 2, an MEP was not able to be elicited in Session 3. The data from this participant for Sessions 1 and 2 were retained for analysis. There were a small number of participant sessions for which LICI stimulations did not elicit a second distinct MEP 100ms following the first MEP, and these sessions were excluded from the analysis (see Tables 1, 2, 3).

**TABLE 1:** Isometric biceps femoris normalised sEM PPA descriptive statistics and test–retest reliability data in response to transcranial magnetic stimulation

Variable	Session 1 (mean ± *SD*)	Session 2 (mean ± *SD*)	Session 3^a^ (mean ± *SD*)	ICC mean (95% CI)	TE mean (95% CI)	CV% (95% CI)	MDC_95_	%SWC
AMT110%	.98 ± .50	1.08 ± .38	.83 ± .32	0.51 (0.04 to 0.84)	0.29 (0.22 to 0.50)	36.82 (25.94 to 66.95)	.80	8.91
AMT120%	1.12 ± .64	1.25 ± .68	1.21 ± .70	0.77 (0.43 to 0.93)	0.43 (0.32 to 0.75)	33.99 (24.01 to 61.33)	1.19	11.78
AMT130%	1.39 ± .77	1.43 ± .84	1.45 ± .84	0.84 (0.57 to 0.96)	0.37 (0.28 to 0.63)	28.71 (20.40 to 51.07)	1.03	11.99
AMT150%	1.54 ± .82	1.59 ± .95	1.94 ± 1.25	0.92 (0.77 to 0.98)	0.42 (0.31 to 0.72)	21.00 (15.05 to 36.56)	1.16	12.82
AMT170%	1.74 ± .91^a^	1.65 ± 1.08	1.81 ± 1.27	0.92 (0.76 to 0.98)	0.34 (0.25 to 0.57)	22.10 (15.88 to 40.38)	.94	13.43
LICI	.67 ± .42^a^	.69 ± .52^a^	.46 ± .23^b^	0.80 (0.39 to 0.95)	0.19 (0.14 to 0.33)	42.48 (28.85 to 95.96)	.53	15.10
SICI	.78 ± .31	.71 ± .27	.57 ± .19	0.69 (0.28 to 0.91)	0.19 (0.14 to 0.31)	26.54 (18.91 to 46.93)	.53	8.11
ICF	1.77 ± .71	1.20 ± .20	1.22 ± .23	0.62 (0.18 to 0.88)	0.32 (0.24 to 0.53)	18.97 (13.63 to 32.83)	.89	5.44
AURC	90.3 ± 42.9^a^	87.6 ± 47.8	94.2 ± 56.4	0.89 (0.68 to 0.97)	18.4 (13.6 to 31.3)	22.67 (16.28 to 41.51)	51.00	11.67

Abbreviations: %SWC, smallest worthwhile change; 95% CI, 95% confidence interval; AMT%, percentage of active motor threshold stimulator output; AURC, area under the recruitment curve; CV%, typical error as a % coefficient of variation; ICC mean, mean Sessions 1 to 2 and 2 to 3 intraclass correlation; ICF, intracortical facilitation; LICI, long‐interval intracortical inhibition; MDC_95_, minimum detectable change at 95% CI; SICI, short‐interval intracortical inhibition; TE, typical error.

^a^ Data representative of only nine participants.

^b^ Data representative of only eight participants.

**TABLE 2:** Eccentric biceps femoris normalised sEMG descriptive statistics and test–retest reliability data in response to transcranial magnetic stimulation

Variable	Session 1 (mean ± *SD*)	Session 2 (mean ± *SD*)	Session 3^a^ (mean ± *SD*)	ICC mean (95% CI)	TE mean (95% CI)	CV% (95% CI)	MDC_95_	%SWC
AMT110%	.87 ± .43	.71 ± .27	.82 ± .32	0.56 (0.09 to 0.86)	0.28 (0.20 to 0.45)	40.58 (28.47 to 74.50)	.78	10.16
AMT120%	1.26 ± .73	1.05 ± .45	1.13 ± .64	0.71 (0.32 to 0.91)	0.39 (0.28 to 0.63)	32.38 (22.92 to 58.17)	1.08	10.13
AMT130%	1.54 ± .90	1.22 ± .63	1.44 ± .94	0.92 (0.76 to 0.98)	0.34 (0.25 to 0.55)	20.29 (14.56 to 35.26)	.94	11.91
AMT150%	1.74 ± 1.19	1.61 ± 1.02	1.71 ± 1.23	0.96 (0.88 to 0.99)	0.29 (0.22 to 0.48)	16.09 (11.60 to 27.63)	.80	13.92
AMT170%	1.91 ± 1.40^a^	1.63 ± 1.14	1.76 ± 1.43	0.90 (0.70 to 0.97)	0.38 (0.28 to 0.65)	29.55 (21.06 to 55.25)	1.05	15.34
LICI	.85 ± .85^b^	.67 ± .32^a^	.45 ± .27^b^	0.86 (0.58 to 0.96)	0.34 (0.25 to 0.59)	42.11 (28.48 to 82.34)	.94	17.69
SICI	.63 ± .21	.68 ± .24	.67 ± .16	0.75 (0.38 to 0.93)	0.11 (0.08 to 0.19)	21.08 (15.11 to 36.71)	.34	7.16
ICF	1.22 ± .48	1.20 ± .36	1.46 ± .29	0.67 (0.25 to 0.90)	0.23 (0.17 to 0.37)	20.13 (14.44 to 34.96)	.63	6.11
AURC	98.44 ± 56.58^a^	80.00 ± 43.55	88.67 ± 59.54	0.94 (0.82 to 0.99)	17.6 (13.0 to 30.0)	16.75 (12.11 to 30.10)	48.78	12.02

Abbreviations: %SWC, smallest worthwhile change; 95% CI, 95% confidence interval; AMT%, percentage of active motor threshold stimulator output; AURC, area under the recruitment curve; CV%, typical error as a % coefficient of variation; ICC mean, mean Sessions 1 to 2 and 2 to 3 intraclass correlation; ICF, intracortical facilitation; LICI, long‐interval intracortical inhibition; MDC_95_, minimum detectable change at 95% CI; SICI, short‐interval intracortical inhibition; TE, typical error.

^a^ Data representative of only nine participants.

^b^ Data representative of only eight participants.

**TABLE 3:** Concentric biceps femoris normalised sEMG descriptive statistics and test–retest reliability data in response to transcranial magnetic stimulation

Variable	Session 1 (mean ± *SD*)	Session 2 (mean ± *SD*)	Session 3^a^ (mean ± *SD*)	ICC mean (95% CI)	TE mean (95% CI)	CV% (95% CI)	MDC_95_	%SWC
AMT110%	1.30 ± .90	1.18 ± .59	1.25 ± .95	0.75 (0.35 to 0.93)	0.52 (0.37 to 0.88)	33.84 (23.98 to 67.39)	1.44	11.30
AMT120%	1.85 ± 1.51	1.42 ± .80	1.65 ± 1.00	0.91 (0.73 to 0.97)	0.49 (0.36 to 0.84)	21.49 (15.40 to 37.47)	1.36	11.77
AMT130%	2.04 ± 1.70	1.63 ± 1.05	1.68 ± 1.23	0.94 (0.81 to 0.98)	0.52 (0.38 to 0.88)	19.59 (14.07 to 33.97)	1.44	13.08
AMT150%	2.08 ± 1.60	1.82 ± 1.27	1.89 ± 1.43	0.96 (0.88 to 0.99)	0.38 (0.28 to 0.65)	15.46 (11.16 to 26.49)	1.05	13.39
AMT170%	2.15 ± 1.59^a^	1.80 ± 1.16	2.04 ± 1.43	0.95 (0.83 to 0.99)	0.41 (0.30 to 0.72)	17.71 (12.79 to 31.92)	1.14	13.15
LICI	.65 ± .56^a^	.63 ± .52^a^	.57 ± .33^b^	0.68 (0.22 to 0.91)	0.29 (0.21 to 0.50)	71.01 (48.30 to 157.14)	.80	18.94
SICI	.66 ± .22	.69 ± .20	.62 ± .23	0.76 (0.40 to 0.93)	0.13 (0.09 to 0.21)	18.61 (13.38 to 32.19)	.36	6.54
ICF	1.18 ± .23	1.10 ± .25	1.13 ± .31	0.65 (0.22 to 0.89)	0.17 (0.12 to 0.27)	16.17 (11.66 to 27.77)	.47	4.81
AURC	120.89 ± 93.18^a^	98.90 ± 61.35	104.44 ± 74.83	0.96 (0.87 to 0.99)	24.0 (17.8 to 40.9)	15.05 (10.90 to 26.90)	66.52	12.66

Abbreviations: %SWC, smallest worthwhile change; 95% CI, 95% confidence interval; AMT%, percentage of active motor threshold stimulator output; AURC, area under the recruitment curve; CV%, typical error as a % coefficient of variation; ICC mean, mean Sessions 1 to 2 and 2 to 3 intraclass correlation; ICF, intracortical facilitation; LICI, long‐interval intracortical inhibition; MDC_95_, minimum detectable change at 95% CI; SICI, short‐interval intracortical inhibition; TE, typical error.

^a^ Data representative of only nine participants.

^b^ Data representative of only eight participants.

### 3.1 AMT and contraction mode intensity during stimulation

The mean stimulator intensity defined as AMT across Session 1 (48% ± 9%), Session 2 (47% ± 9%) and Session 3 (46% ± 8%) demonstrated excellent reliability (mean ICC, .95; 95% CI, .84 to .99; TE mean, .26; 95% CI .19 to .44; %TE mean, 5.13; 95% CI, 3.74 to 9.93; MDC_95_, .72). Pre‐stimulus rmsEMG prior to stimulations for all trials was 4.93% ± .89% of maximal rmsEMG.

### 3.2 Isometric contractions

**FIGURE 2:**
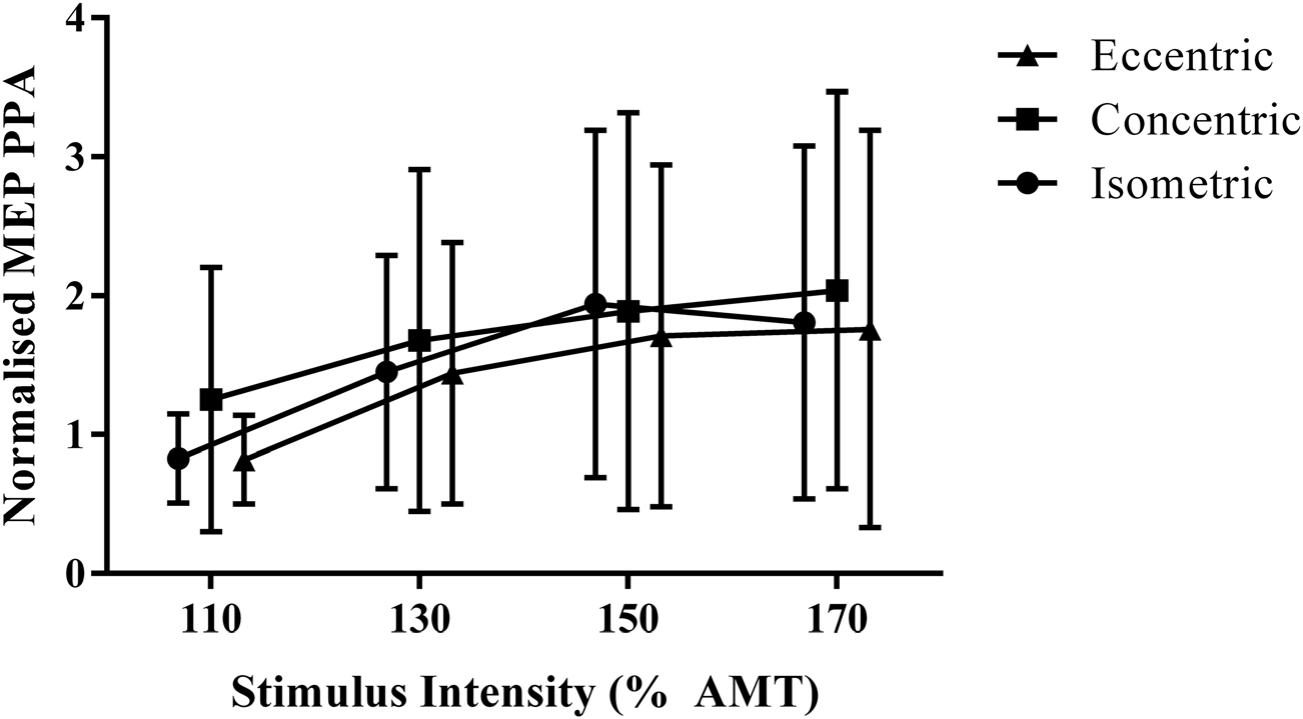
Transcranial magnetic stimulation (TMS) stimulus–response curve of the biceps femoris during isometric, concentric and eccentric contractions. Data were derived from participants (*n* = 9) during their third and final testing session. For all contraction modes, the contraction intensity prior to the stimulation was 5% ± 2% of maximal biceps femoris root mean squared electromyography (rmsEMG) during an isometric knee flexor contraction. Peak‐to‐peak amplitude (PPA) of the motor evoked potential (MEP) was, normalised to the rmsEMG derived from a maximal isometric voluntary knee flexor contraction. AMT%, the stimulation intensity as a % of active motor threshold. Stimulus response curves = surface electromyography responses collected across all contraction modes by delivering stimuli at each of 110%, 130%, 150% and 170% of AMT

**FIGURE 3:**
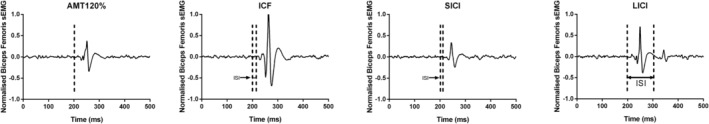
Exemplar participant motor evoked potential data during isometric knee flexion contraction at AMT120%, ICF, SICI and LICI. Dashed lines indicate when stimuli were applied. AMT120%, the stimulation intensity of 120% active motor threshold; ICF, intracortical facilitation; SICI, short‐interval intracortical inhibition; LICI, long‐interval intracortical inhibition; ISI, interstimulus interval; sEMG, surface electromyography

The AURC and normalised MEP PPA recorded at AMT120%, AMT130%, AMT150% and AMT170% showed excellent reliability (ICC range, .77 to .92) (Table 1 and Figures 2 and 3). AMT110% showed fair reliability (ICC, .51) (Table 1). The paired‐pulse outcomes, SICI, LICI and ICF, demonstrated good to excellent reliability under isometric conditions (ICC range, .62 to .80) (Table 1).

### 3.3 Eccentric contractions

Single‐pulse outcomes measured during eccentric contractions which demonstrated excellent reliability include the AURC and normalised MEP PPA measured at AMT130%, AMT150% and AMT170% (ICC range, .90 to .96) (Table 2 and Figure 2). Normalised MEP PPA recorded at AMT120% was shown to have good reliability (ICC, .71), whereas fair reliability was found for AMT110% (ICC, .56) (Table 2). The paired‐pulse outcomes, SICI, LICI and ICF, demonstrated good to excellent reliability under eccentric conditions (ICC range, .67 to .86) (Table 2).

### 3.4 Concentric contractions

Single‐pulse outcomes measured during concentric contractions, including the AURC and MEP PPA recorded at AMT110%, AMT120%, AMT130%, AMT150% and AMT170% all demonstrated excellent reliability (ICC range, .75 to .96) (Table 3 and Figure 2). During concentric contractions, SICI demonstrated excellent reliability (ICC, .76) (Table 3). However, LICI and ICF were shown to have poor between‐session reliability (ICC range, .65 to .68) (Table 3).

## 4 DISCUSSION

To the authors' knowledge, this is the first study to assess the reliability of TMS derived measures of corticospinal properties from the biceps femoris during isometric, concentric or eccentric contractions. This study found that single‐pulse TMS outcomes resulted in fair to excellent reliability across all contraction modes, with higher stimulation intensities (i.e., AMT130%, AMT150% and AMT170%) consistently resulting in excellent reliability. In addition, the AURC also displayed excellent reliability across all contractions. With respect to paired‐pulse stimuli, the reliability was fair to excellent; however, there was larger variation across contraction modes.

Other lower limb muscles, including the vastus lateralis (O'Leary et al., 2015; Temesi et al., 2017), vastus medialis (Leung et al., 2017; Temesi et al., 2017), rectus femoris (Sidhu et al., 2009; Temesi et al., 2017), gastrocnemius (Fisher et al., 2014) and the tibialis anterior (Fisher et al., 2014; Tallent et al., 2012; van Hedel et al., 2007) have had the reliability of TMS derived measures of corticospinal function investigated previously, most commonly during isometric contractions. Previous work assessing corticospinal excitability using single‐pulse TMS during isometric contractions in the vastus lateralis (O'Leary et al., 2015; Temesi et al., 2017), vastus medialis (Leung et al., 2017; Temesi et al., 2017), tibialis anterior (Fisher et al., 2014) and gastrocnemius (Fisher et al., 2014) have demonstrated fair to excellent between‐session reliability (ICC range, .63 to .94), which is in agreement with the findings of the current study. Furthermore, other literature that have utilised paired‐pulse TMS assessments during isometric contractions in lower limb muscles to determine SICI have found fair to excellent reliability in the vastus lateralis (ICC range, .53 to .93) (O'Leary et al., 2015; Temesi et al., 2017), vastus medialis (ICC range, .78 to .87) (Leung et al., 2017) and rectus femoris muscles (ICC range, .56 to .84) (Temesi et al., 2017) whereas SICI consistently showed good to excellent reliability in the current study across all muscle actions. Observations of good to excellent reliability in LICI in this study (ICC range, .68 to .86) was slightly better in comparison with previous literature utilising paired‐pulse TMS in the vastii and peroneus longus which demonstrated fair to excellent between‐session reliability (ICC range, .47 to .93) (O'Leary et al., 2015; Temesi et al., 2017). In contrast, observations of good reliability for ICF in the current study (ICC range, .62 to .67) were marginally superior to similar literature which found fair to good reliability in the vastii (ICC range, .51 to .61) (O'Leary et al., 2015). There is limited capacity to compare the results of this study to similar literature as there is only one other study which has assessed the reliability of single‐ and paired‐pulse measures during concentric and eccentric contractions in a lower limb muscle (Tallent et al., 2012).

Single‐ and paired‐pulse outcomes assessed within this study demonstrated variability in the values measured from session to session. Therefore, it is important to establish whether changes in such outcomes can be detected and interpreted confidently despite this variability. A measure is considered sensitive if it can detect the SWC. This means that the measure would be considered sensitive enough to detect between‐session changes if the CV% is less than the SWC. While many of the outcomes assessed in this study demonstrated acceptable reliability, none were sensitive enough to detect the SWC. This poses a challenge for studies utilising TMS outcomes from the biceps femoris to measure changes in corticospinal function, as these data suggest that a moderate or greater effect size change is required to be able to deduce, with a relative degree of certainty, that an actual change has occurred. Substantial variation was found in the current study, with single‐pulse variables reporting a CV% of 15% to 41%, whereas SICI, LICI and ICF had CV% of 19% to 27%, 42% to 71% and 16% to 20%, respectively. Other lower limb literature have made slightly better findings to the current study with CV% for single‐pulse corticospinal excitability measures of between 5% and 26% (Fisher et al., 2014; O'Leary et al., 2015; Temesi et al., 2017), whereas the CV% for paired‐pulse measures appears similar or somewhat worse compared with the current work (SICI, LICI and ICF demonstrated CV% of 10% to 29%, 12% to 72% [O'Leary et al., 2015; Temesi et al., 2017] and 16% to 18% [O'Leary et al., 2015], in prior work, respectively). Notwithstanding, the current data should provide useful information for future work seeking to utilise corticospinal and intracortical measures using various stimulus intensities in other lower limb muscle groups.

The TMS outcomes utilised by the current study could feasibly be used to investigate alterations to injured populations such as those with a history of hamstring strain injury. Multiple studies have detected reduced sEMG activity of the biceps femoris during maximal contractions in those with a history of hamstring strain injury (Opar et al., 2013). Deficits in nervous system activity may have implications for reinjury, as *in‐situ* experiments have demonstrated reductions in the amount of energy absorbed when comparing submaximal and maximally activated muscle during lengthening (Mair et al., 1996). However, observations of altered nervous system function following muscle injury are restricted to measures of sEMG amplitude (Opar et al., 2013) or voluntary activation (via the twitch interpolation method) (Buhmann et al., 2020) during maximal strength assessments. Despite sEMG providing information regarding the timing and degree of muscle excitation (Vigotsky et al., 2017), using TMS in addition to sEMG adds the ability to detect the site of alterations in corticospinal function using paired‐pulse measures such as SICI. Should higher SICI, or greater inhibition local to the M1, be observed in those with a history of strain injury, such information could be utilised in the optimisation of interventions for rehabilitation. For example, while SICI has shown to be responsive to strength training interventions (Kidgell et al., 2015; Latella et al., 2016), it is reduced to a greater extent following eccentric strength training when compared with concentric interventions (Kidgell et al., 2015). The acceptable reliability shown in several variables in this study demonstrates promising application in tracking alterations in indicators of corticospinal excitability, intracortical inhibition and facilitation within the context of injury, immobilisation, rehabilitation or interventions.

There are several methodical variations from the current study that could be considered for future work. This study utilised a common approach of normalising MEP PPA collected during SICI and ICF assessments to MEP PPA data collected from a separate AMT120% test stimulus (Kidgell et al., 2015; Latella et al., 2016). However, this study found a larger range in the reliability of the AMT120% assessment (ICC range, .71 to .91) compared with AMT130% (ICC range, .84 to .94), and the latter has also been used to normalise SICI and ICF data in upper limb muscles (Garry & Thomson, 2009). Should this method be used in future research to detect changes in SICI following training interventions or injury, utilising intensities with lower measurement error, or higher stimulus intensities such as AMT130%, may provide a superior capacity to detect changes in within‐subject physiological parameters. Higher test stimulation intensities have been shown to elicit greater SICI PPAs and lower stimulation intensities can fail to detect SICI (Garry & Thomson, 2009). Further clarification is needed as to what the optimal test stimulus intensity may be to measure SICI, as stimulation intensities which are too high or low may tend to either underestimate or fail to detect cortical inhibition (Garry & Thomson, 2009). Alterations in the intensity of contraction may also influence the reliability of TMS outcomes from the biceps femoris. The current study utilised a 5% ± 2% of maximal rmsEMG contraction target to standardise cortical excitability across assessments. While it is difficult to make comparisons with studies which set contraction intensity targets using a %MVC torque target, greater reliability in single‐pulse cortical excitability assessments is evident at higher contraction intensities (van Hedel et al., 2007) and should be considered in future investigations. Poorer reliability was found in the current study in LICI compared with similar literature, which may be due to many paired‐pulse stimulations failing to induce two distinct or detectable MEP PPAs (Table 3). Further improvements in the within‐ and between‐participant variability of paired‐pulse outcomes may also be observed by individualising conditioning stimulus intensities to optimise evoked inhibitory or excitatory responses (Orth et al., 2003). At least 10 stimuli per measure are recommended for single‐site TMS measuring corticospinal excitability across multiple sessions (Cavaleri et al., 2017). While there is no evidence to suggest significant increases in between‐session reliability with increasing numbers of stimulation (Cavaleri et al., 2017), wide confidence intervals in upper and lower limb TMS studies indicate more stimulations may be efficacious. However, prolonged TMS assessments are both physically and mentally demanding on the participant (van de Ruit et al., 2015). Additionally, corticospinal excitability fluctuates with participant arousal and concentration (Classen et al., 1998) which may vary across prolonged assessments. It is feasible that variation across a prolonged testing protocol, stimulation parameters and muscle activity could vary these outcomes.

There are limitations within the current study which require consideration. First, between‐session variability in the placement of sEMG sensors may have occurred, despite the consistent use of SENIAM guidelines. Similarly, changes in TMS coil positioning and orientation may have occurred between stimulations within a session, which has been shown to alter TMS outcomes at a participant, but not group level (de Goede et al., 2018). Second, participants included in this study were largely young, healthy males, which may limit the generalisability of the reliability of the method to populations exhibiting pathology. Third, TMS studies often normalise MEP PPA data to maximal M‐wave data obtained during electrical stimulation of a peripheral nerve which innervates the target muscle (O'Leary et al., 2015). The advantage of using peripheral muscle excitability, or M‐waves, to normalise data is that these values remain largely unchanged across sessions or interventions (Kidgell et al., 2015). However, this study normalised MEP PPA data to rmsEMG data obtained during an isometric MVC which is highly reliable across sessions (Bussey et al., 2018). Electrical stimulation of the sciatic nerve or hamstring muscles is associated with significant discomfort or pain (Kirk et al., 2018) due to the larger stimulation intensities required to elicit maximal responses compared with other structures such as the femoral nerve. Furthermore, the test–retest reliability of electrical stimulation of the sciatic nerve or hamstring muscles is unknown. Additionally, the use of rmsEMG obtained during an isometric MVC to guide eccentric and concentric pre‐stimulation rmsEMG is a limitation of this study. While all trials which were outside of 5 ± 2% rmsEMG were discarded, spinal and supraspinal neural control during dynamic contractions is altered (Aagaard, 2018), which may have influenced the MEP responses observed. For example, when compared with maximal isometric contractions, maximal eccentric contractions are associated with decreased corticospinal excitability (Aagaard, 2018).

In conclusion, this study found fair to excellent reliability across all contraction modes for single‐pulse TMS derived measures of corticospinal excitability from the biceps femoris, as well as for the AURC. The paired‐pulse variables demonstrated good to excellent reliability across contraction modes. Large CV% values, which were always greater than the SWC, were found across all variables and muscle actions. This highlights that changes need to be more than a small effect size if TMS derived measures of biceps femoris corticospinal excitability, intracortical inhibition or facilitation are to detect physiological change at an individual level.

## 5 PERSPECTIVE

While TMS is widely utilised in the muscles of the lower limb, the reliability of assessing the corticospinal excitability and intracortical inhibition and facilitation of the biceps femoris is yet to be determined. As the biceps femoris is the most commonly injured of the hamstring muscle group, further research into its function is needed to attempt to reduce the incidence of injury. This study demonstrated that single‐pulse and paired‐pulse TMS outcomes of the biceps femoris were reliable across concentric, eccentric and isometric contraction modes. This is the first time both single‐ and paired‐pulse TMS measures have been assessed across all contraction modes in a muscle of the lower limb. As such, the findings can be used to guide future work aiming to utilise repeated TMS measures to assess corticospinal function and intracortical inhibition of the biceps femoris, such as before and after training interventions or fatigue inducing protocols.

## AUTHOR CONTRIBUTIONS

**David A. Opar:** Conceptualization; data curation; formal analysis; investigation; methodology; project administration; resources; software; supervision; validation; visualization; writing – original draft; writing – review and editing. **Dawson J. Kidgell:** Conceptualization; data curation; investigation; methodology; resources; software; supervision; validation; visualization; writing – original draft; writing – review and editing. **Paul J. Tofari:** Conceptualization; data curation; formal analysis; investigation; methodology; project administration; resources; software; supervision; validation; writing – original draft; writing – review and editing. **Joel D. Presland:** Conceptualization; data curation; formal analysis; investigation; methodology; project administration; resources; software; validation; visualization; writing – original draft; writing – review and editing. **Ryan G. Timmins:** Investigation; project administration; resources; supervision; visualization; writing – original draft; writing – review and editing.

## CONFLICT OF INTEREST

The authors have no conflicts of interest to declare.

### PEER REVIEW

The peer review history for this article is available at https://publons.com/publon/10.1111/ejn.15868.

## Data Availability

The data that support the findings of this study are available on request from the corresponding author. The data are not publicly available due to privacy or ethical restrictions.
